# Development and Introduction of Fexinidazole into the Global Human African Trypanosomiasis Program

**DOI:** 10.4269/ajtmh.21-1176

**Published:** 2022-03-15

**Authors:** Olaf Valverde Mordt, Antoine Tarral, Nathalie Strub-Wourgaft

**Affiliations:** Drugs for Neglected Diseases initiative, Geneva, Switzerland

## Abstract

In this article, the authors show the strategy used to streamline the introduction of fexinidazole, the first all oral treatment of human African trypanosomiasis (HAT) caused by *Trypanosoma brucei gambiense. *The dose range was determined in phase 1 studies and a significant food effect was observed, which was tested with field-adapted meals. The pharmacokinetic profile required definition of a higher loading dosage for the first 4 days and administration of the daily dose together with a typical local meal to optimize product absorption and rapidly achieve drug steady state. This allowed for a combined phase II/III pivotal study directly after phase I trials. Partnerships with highly engaged actors from endemic country control programs and international research institutions started early through the HAT platform, building on an agreed target product profile (TPP), establishing a regulatory plan early and transparently including endemic countries in the research and data flow. A key element that enabled a quick start to access activities was preparing for World Health Organization guidelines early and starting the process prior to registration. Distribution plans were identified and supply was established from the start, by taking advantage of the existing supply agreement between the producers of all HAT drugs (Sanofi and Bayer) and the WHO. Pharmacovigilance and phase 4 studies were nested into wider implementation activities. Targeted sequential introduction into national programs was prioritized, based on medical need and epidemiologically updated information.

## INTRODUCTION

### Background/landscape of human African trypanosomiasis.

Human African trypanosomiasis (HAT), or sleeping sickness, is a disease caused by two subspecies of the unicellular flagellated parasite *Trypanosoma brucei: T. b. rhodesiense* (r-HAT) and *T. b. gambiense* (g-HAT); the disease is transmitted through the bite of a tsetse fly. ^1^ Infection occurs exclusively in sub-Saharan Africa in two distribution areas, in the east of the continent for r-HAT and in central and western countries for g-HAT. g-HAT carries the highest burden. The classic disease-defining symptom is the rupture of the sleep-wake cycle into small time periods, causing bouts of day sleep and night insomnia. If untreated, the patient deteriorates into an almost inevitable death—within months to years for g-HAT and even faster for r-HAT. ^2^ Clinically, r-HAT evolves more rapidly but has a similar pattern to g-HAT, beginning with initial hemolymphatic dissemination from the bite with general symptoms (e.g., irregular fever, headache, weakness, and pruritus) and then—after crossing the blood–brain barrier—provoking a meningoencephalitic infection with more specific neuropsychiatric and endocrinological symptoms.

Without treatment, patients will invariably progress from the first to the second stage of the disease, which is when parasites have invaded the central nervous system. Therefore, treatment at that stage needs to cross the blood–brain barrier effectively.

Melarsoprol, an arsenic-based treatment, was introduced in 1949 for stage 2 HAT. This injectable drug, described as “fire in the veins” by patients, was highly effective and lifesaving, but is associated with encephalopathy in 10% of patients, and is directly lethal in 5% of patients. ^3^ This risk was well known to communities: although it could save lives, treatment with melarsoprol was also perceived as a threat. Researchers later found that eflornithine, which was initially developed as an anticancer treatment, had antitrypanosome activity; since it easily crosses the blood–brain barrier, it was repurposed for stage 2 HAT. A regimen of four slow infusions every 6 hours for 2 weeks showed very good efficacy. Eflornithine is not associated with lethal toxicity, but it is still not optimally tolerated. More importantly, physical access to treatment was limited for many because of the need for hospitalization in specialized centers and the heavy demand it made on health resources.

In the early 2000s, Doctors Without Borders/Médecins Sans Frontières (MSF), Epicenter, and later the Drugs for Neglected Diseases *initiative* (DND*i*) initiated a phase 3 study comparing a shorter combined regimen of eflornithine (only two infusions per day for 7 days) plus nifurtimox, an oral treatment already registered for Chagas disease (caused by American trypanosomes), given three times a day for 10 days. This shorter treatment is known as nifurtimox-eflornithine combination therapy (NECT). NECT was a simpler regimen that showed similar efficacy to eflornithine alone as well as good safety in stage 2 HAT. It therefore in 2009 became the reference for the treatment of stage 2 HAT. Nifurtimox-eflornithine combination therapy was subsequently added to the World Health Organization (WHO) Essential Medicines List and, following extensive training by WHO and national sleeping sickness control programs (NSSCPs), was rapidly deployed in countries.

Meanwhile, little progress was made on treating patients at the earlier stage of the disease, where the first-line treatment was mostly intramuscular pentamidine. (Pentamidine is not suitable for use in stage 2 disease, as it does not reach sufficient concentrations in the brain. ^4^) Given the complexity of NECT, the recommendation to treat stage 1 patients with pentamidine remained, and only stage 2 HAT was treated with NECT. To determine whether patients were in stage 1 or 2, a lumbar puncture was necessary to discern whether parasites had invaded the central nervous system; the parasites would either be detected directly by microscopy or indirectly by raised levels of white blood cells in the cerebrospinal fluid, which indicate inflammatory changes. ^5^

In 1960, around the time of independence of most HAT-endemic countries, the reported number of HAT cases was low. The perceived low importance of sleeping sickness, new conflicts, and the challenge of building independent health systems led to a reduced effort to control trypanosomiasis; a subsequent slow but constant increase of cases resulted in an epidemic surge by the end of the 1980s. ^6^ Few international nongovernmental organizations were involved in HAT control activities—MSF being the most active of these—while the NSSCPs were poorly staffed and funded. At the turn of the twenty-first century, WHO developed a program to reduce disease transmission, reinforcing the NSSCPs and partnering with international stakeholders, including the two companies that produced all the drugs for HAT, Sanofi, and Bayer. The not-for-profit foundation DND*i* was created in 2003 with the aim of contributing to the development of drugs for neglected patients, and HAT was one of the diseases initially targeted. Human African trypanosomiasis was selected in light of the field experience of medical doctors from MSF—one of the DND*i* founding institutions—who were faced with the dilemma of using a lifesaving drug, melarsoprol, which killed 5% of patients. Drugs for Neglected Diseases *initiative* joined Epicenter and MSF in 2005 with the aim of improving treatment of HAT by completing the development of NECT by 2008. ^7^

Case searching steadily improved and the number of reported cases decreased, which, in 2012, enabled WHO to target HAT for elimination. ^8^ By the end of January 2012, a group of neglected tropical disease stakeholders launched the London Declaration, which aimed to support the recently defined WHO road map for neglected tropical diseases, helping to accelerate progress toward elimination or strongly reduce the prevalence of most neglected tropical diseases, including HAT. ^9^ Human African trypanosomiasis case numbers have reduced from a peak of 37,991 cases reported in 1998 to less than 1,000 cases reported annually since 2018.

### An opportunity for innovation.

The HAT platform, founded in August 2005, is constituted of key HAT stakeholders, such as NSSCPs, research centers in the most endemic countries, and international research partners. The World Health Organization participates as an observer. Following the results from NECT clinical research, the HAT platform updated the target product profile (TPP) for HAT drugs in 2010. One of the major requirements was a stage-independent and oral-only treatment. An oral treatment would be better adapted for use in the contexts where most HAT patients live: it would enable HAT treatment to be delivered on an outpatient basis, and require hospitalization only for those needing specialized management or monitoring.

After identification of the nitroimidazole family as potentially active against HAT, an extensive compound mining effort, searching among more than 700 nitroheterocycles, resulted in the rediscovery of fexinidazole. This broad spectrum antiparasitic was initially developed in the 1970s but later abandoned. Its in vivo activity against HAT had been identified in 1983, ^10^ and in 2006, DND*i* selected fexinidazole for further preclinical development. ^11^ The phase 1 clinical program started in September 2009 and was completed in 2012. Healthy human subjects received single ascending doses, single fixed doses, or multiple ascending doses of orally administered fexinidazole according to different dosing regimens. The relative bioavailability of different oral formulations of fexinidazole, and the impact on bioavailability of different types of food, including meals similar to those generally available to affected populations, were also investigated. ^12^ The dosage was determined during phase 1, thus avoiding an additional phase 2 dose-ranging study, paving the way to a pivotal phase 2/3 clinical trial comparing the efficacy and safety of fexinidazole to NECT in advanced-stage adult patients with evidence of parasites. The first patient started treatment on October 7, 2012, in the Democratic Republic of the Congo (DRC). ^13^

### Accelerating timelines from development to introduction.

Several factors enabled the acceleration of the fexinidazole timeline from development to introduction. First, work in partnership with all stakeholders to achieve and maintain alignment on fexinidazole’s medical value started early, at the time of the TPP, ensuring that a new treatment would respond to the needs of the patients and have a feasible rollout. Second, manufacturing and distribution were supported by an agreement between DND*i* and Sanofi, signed in 2009. Third, a regulatory strategy was devised to ensure quick registration in endemic countries and hence rapid access to patients. Sanofi and DND*i* selected the combined approach of requesting 1) a scientific opinion from the European Medicines Agency (EMA) Committee for Medicinal Products for Human Use through the Article 58 procedure (recently renamed as EU-M4all) for products intended to prevent or treat diseases of major public health interest and for use exclusively outside the European Union and 2) inclusion of the product into the Essential Medicines List and WHO prequalification on the basis of assessments and inspections conducted by EMA. The positive opinion from the Committee for Medicinal Products for Human Use was the basis for facilitating registration in DRC and Uganda and subsequent approval by the Ministries of Health of the remaining g-HAT-endemic countries. Finally, the study progress and the access strategy were discussed regularly at WHO HAT stakeholder meetings and by the specific working group on integration of new tools.

### Working in partnership.

The main objective of the HAT platform was to facilitate the research environment by increasing technical capacity and to prepare for future access to the treatments developed through local research. ^14^ Funding provided through DND*i* enabled the HAT platform to organize training sessions, engage local authorities from Ministries of Health to facilitate access to the new treatment, and promote pharmacovigilance. Annual meetings were frequently combined with scientific events and were useful for sharing new research developments in the HAT field among the partners. As a result, the HAT platform had a fundamental role in keeping all relevant partners informed about the progress of the clinical research. The HAT platform board had a central role both in defining the TPP that led to the selection and development of fexinidazole and in keeping the governments of endemic countries aware of the progress and results of the clinical trials.

In 2009, before starting phase 1 studies, DND*i* and Sanofi reached an agreement to jointly develop fexinidazole, with DND*i* in charge of the clinical development and Sanofi responsible for product registration, industrial development, and further regular supply, if successful. The DRC NSSCP, and other NSSCPs from the HAT Platform, were key partners throughout the process, from study development and implementation to fexinidazole training and finally rollout and surveillance. In fact, the NSSCPs of DRC, Central African Republic, and Guinea led the choice of study hospitals, based on the reported HAT endemicity, and managed the research teams for each hospital via a coordinating investigator who ensured stability and harmonized the trial activities by providing regular supervision. All partners were regularly updated and consulted via regular meetings organized by WHO and HAT stakeholders, as well as meetings of a specific working group focused on development of new tools, which discussed diagnostics and treatment needs and advances.

### Working with regulators.

The EU-M4all procedure of the EMA has the advantage of being in collaboration with WHO and regulators from targeted HAT-endemic African countries, which in the case of fexinidazole were from DRC and Uganda. The aim of the procedure is to streamline review of the dossier once it is presented to the countries. Two scientific advice meetings were arranged to review the phase 2/3 pivotal trial protocol and to propose two complementary cohort studies to widen the populations studied. Before the study, DND*i*, with the support of WHO, conducted a joint ethics prereview ^15^ in which various experts from endemic and nonendemic countries participated, resulting in an extensive and collaborative assessment. The final submission to the EMA included the full product dossier, which was prepared by DND*i* and Sanofi project teams; this received a fee exemption and accelerated review timelines. A positive scientific opinion was granted by the EMA Committee for Medicinal Products for Human Use on November 15, 2018, after two rounds of questions and answers.

The marketing authorization approval in DRC was granted in 2018, 6 weeks after the positive opinion from the EMA Committee for Medicinal Products for Human Use.

### Preparing for introduction.

The choice of target countries and priority actions was based on the prevalence of reported HAT cases and started with the DRC, which reported 82.5% of all HAT cases in the last decade (2011–2020). The process for country adoption began after fexinidazole received authorization for use in DRC. Subsequently, WHO engaged the Ministries of Health of all g-HAT-endemic countries to give a special authorization for use based on the DRC registration. By July 2021, this authorization had been obtained from seven additional countries. The initiation of the WHO guidelines development process early on, as soon as the study results had been published, 1 year before registration facilitated the accelerated pace toward introduction and for the guidelines to be released in August 2019. The participating countries reviewed and adapted these guidelines and adopted national ones. The WHO HAT team then organized two sessions to train trainers. Through the HAT Platform, DND*i* supported the NSSCPs to extend the training to all staff from 250 selected health structures that would deliver HAT treatment.

The EMA requested Sanofi to conduct a post approval safety study, and data are now being gathered by the NSSCPs, with technical support from WHO to provide additional safety information to that which was obtained from the relatively low number of participants included in the clinical trials. This new data on real-life safety and effectiveness of fexinidazole will be a retrospective analysis over 3 years, from March 2020, when the product was first supplied to DRC, until February 2023, and including all HAT-endemic countries that have detected cases, once fexinidazole is delivered to each of them. In addition, a simplified pharmacovigilance reporting system and tools were prepared, provided to all NSSCPs, and included in the training of prescribers. Drugs for Neglected Diseases *initiative’s* support to pharmacovigilance reporting is actively concentrated in the most endemic or difficult-to-reach countries and areas. Regular supervisory visits have been organized in the DRC and will be proposed for Guinea, Central African Republic, Angola, and South Sudan.

Human African trypanosomiasis treatment distribution was easy to organize. This was the result of agreements between Sanofi and WHO, in place since 2001, to provide pentamidine, eflornithine, and melarsoprol to the endemic countries in a centralized way and at no cost. A similar agreement exists with Bayer for nifurtimox and suramin. Each country provides its epidemiological data and discusses with the WHO team its needs for the coming year. The World Health Organization informs Sanofi and Bayer of the collective needs, then both companies forecast manufacturing needs and provide the drugs to the MSF Logistique supply center, which has agreed to store the drugs and organize the international aspects of drug distribution. With the authorization of WHO, the MSF Logistique supply center can also provide fexinidazole to international or national nongovernmental organizations who have justified the need. Each country then requests a specific supply for all drugs to the WHO HAT team, who then requests the MSF Logistique supply center to send the requested drugs to the relevant NSSCP. The much smaller volume of materials needed for fexinidazole treatment compared with NECT (which requires infusion sets and bags) simplifies the supply process and reduces transport costs. The fact that fexinidazole could fit into this previously existing system removed any potential delays related to setting up new systems for procurement and supply.

### Challenges associated with accelerating introduction.

The introduction of NECT was rapid for two main reasons. First, it replaced the highly toxic melarsoprol as a first choice for treatment, and second, because of the need for intravenous infusions of eflornithine, the target health structures for NECT were general reference hospitals. Nifurtimox-eflornithine combination therapy’s better safety profile, quick addition to WHO’s Essential Medicines List, training for prescribers and treating nurses, and use of existing donation and distribution programs were the operational elements for success. The main strength of fexinidazole compared with NECT has been the transformation of g-HAT into an “ordinary” disease, with no requirement for lumbar puncture or to travel for specialized hospital care, except for patients with advanced stage disease (who require NECT) or children under 6 years old or weighing less than 20 kg (who remain ineligible for fexinidazole treatment). Only by simplifying case management procedures we can transfer the responsibility of care from a vertical program into the general health care structures, allowing the disease to be integrated into the standard primary health care activity package. The next challenge, then, was of introducing HAT treatment into regular peripheral health structures that had no previous experience in diagnosing or treating HAT. A large training program directed at the staff of 250 selected peripheral centers in the targeted countries had to be designed to familiarize them with the clinical symptoms, diagnostic algorithm, and treatment administration and monitoring requirements.

To reach over 500 remotely based personnel from the chosen health structures, cascade training was designed in close coordination with the WHO HAT team, the NSSCPs, and DND*i*. The first step, taken by WHO, was to train the trainers from the endemic countries in the clinical characteristics of the disease, its treatment, and pharmacovigilance. These trainers were subsequently supported by DND*i* resources to organize cascade training of chosen practitioners at the national level, starting in the most endemic countries, with particular focus on the DRC, as it has the largest and most complex needs because of the high relative prevalence of the disease in comparison with the rest of the endemic countries. A related challenge was the need to accompany direct fexinidazole training with improvement of screening and diagnostic capacity for the selected peripheral health structures. A parallel training program directed toward laboratory personnel at these health structures was designed and implemented in DRC. Finally, the poor status of the health system structures themselves meant that up to 30 peripheral health structures in DRC had to either be built or rehabilitated and then equipped to diagnose HAT.

Another challenge specific to HAT is that its reduced prevalence and nonspecific symptoms often leads to delays in health-seeking behaviors, which in turn result in a long and slow pathway to correct diagnosis of infected individuals. Additionally, there is a low level of awareness of the risks of undetected disease. Therefore, it became clear that social science interventions were needed. Ethnographic studies qualitatively assess communities’ perception of the disease, as well as related health-seeking behaviors. ^16^ A new study attempted to elucidate the main myths related to disease origin and treatments as well as cultural interdictions that could delay community members’ awareness of the disease, suspicion of infection, and search for adequate sources of help. This study (to be published) brought relevant insights on how to design information, education, and communication tools and was used to guide a campaign to create community awareness about HAT and current treatment options.

Yet another challenge to the introduction of fexinidazole was that it required a complex preliminary setup, including a three-step process that started by generating treatment guidelines and training the trainers. Then followed the adoption of the new oral treatment, including translating WHO guidelines into national guidance. Launching the supply required both formal authorization of the use of fexinidazole by those governments that had not approved a regular market authorization and training of staff involved in fexinidazole administration. Finally, the national authorities requested the drugs, which were donated by Sanofi, authorized by WHO, and supplied through MSF Logistique. Fexinidazole was introduced into different countries sequentially, starting in DRC. Since the supply is centralized by WHO, which forecasts production according to national reports, and there is a small number of patients to be treated, there have been no relevant product supply issues. In DRC, because of the large geographical distribution of HAT and the small number of cases in each focus, the NSSCP decided to store fexinidazole at the provincial coordination level, enabling the drug to be sent to the selected treatment center when new cases were identified. Scale-up activities were severely disrupted in places by the COVID-19 pandemic, which arrived at the time of the cascade training and delayed several other activities. In addition, COVID-19 had a critical impact on screening activities, which resulted in fewer patients screened and only 663 diagnosed in 2020, which was lower than expected. ^17^

### Looking ahead on the road to impact.

By June 2021, 200 patients had been treated with fexinidazole in four countries. Most of them were treated in DRC but also, in Chad, Guinea, and Central African Republic. Since its introduction in each country/province, the treatment coverage with fexinidazole has been slightly above 50% of all cases. This coverage is directly in line with fexinidazole being indicated as the first-line treatment, except for 1) patients with neurological symptoms and over 100 white blood cells per µL of cerebrospinal fluid who require the use of NECT or 2) children less than 6 years old or weighing less than 20 kg who require pentamidine or NECT according to the severity of the disease. New countries with lower endemicity are being trained and will receive the new treatment once the requirements (e.g., guidelines, training, national approval, and formal request to WHO) are fulfilled. The potential impact of fexinidazole depends on local case-finding capacities. Information, education, and communication activities in endemic areas remain important to create awareness in communities and encourage potentially affected patients to get diagnosed and treated; these activities also clarify and dispel existing myths about the disease and the remaining fear from the toxic arsenic-containing treatment. Training of prescribers needs to be continually available as the existing health staff may turnover, and new staff arriving to the identified treatment centers may not be aware of HAT and the appropriate treatments.

## DISCUSSION

The disease is on its way to elimination. The number of detected cases has reduced from 38,000 in 1998 to less than 1,000 annually since 2018. The downside of this great success is that young health professionals are not interested in specializing in HAT and the existing expertise is fading away as workers involved in HAT diagnosis and treatment retire. Governments of HAT-endemic countries will reduce their attention on combating this disease as they have many urgent conflicting priorities in healthcare and little dedicated budget. International attention and financial support may fade if elimination has not occurred by the target date for absence of cases in 2030. This decrease in priority, attention, and resources as cases continue to decline could put the goal of elimination at risk.

### Lessons learned for introduction of new interventions.

Most countries in sub-Saharan Africa with endemic diseases have weak health systems, difficult physical access to specialized treatment structures, and high costs, even if the diagnosis and treatment of a condition is given free at the point of delivery. It was essential to consider this context from the start of the development and access activities and examine the local blocking points to provide tailored support that would increase accessibility.

Training cannot be delivered just once but requires careful follow-up to detect and correct weaknesses and to support newly deployed health personnel. Training cannot only be technical, centered on specific trial good clinical practice or medical needs; training must include all aspects related to project management, including financial management, supply logistics, timely requests to renew consumables, human resources management, and issues related to strict respect of standard precautions related to hygiene and waste management.

Energy and telecommunications needs must be carefully assessed, and bottlenecks solved. It is highly advisable to have a backup solution on hand for most elements at risk, for example, by combining different sources of electricity generation or alternative methods of telecommunication.

The research team must communicate in two directions that are not always considered relevant: with the communities and with local authorities. The communities must understand the reasons for the research and the impact the results may have on their health. Once development is completed, the research team needs to share results with the community to generate demand for the new tool. The research team also needs to communicate with local authorities, and this communication needs to go beyond the usual ethical and regulatory review processes, as authorities will need to understand that moving staff around during a clinical trial or after a training effort will directly affect the quality of services provided. Furthermore, the research team must have a clear understanding of the needs for, and perceptions and expectations of, the new development. Social science research on people’s prevailing understanding of the disease, existing beliefs, its treatment, and health-seeking behavior will be helpful, especially during the access phase of the project.

Despite the challenges, there are clear advantages to compressing development and introduction timelines to rapidly bring an improved solution to a problem. There are two main aspects to trying to reduce development and access timelines. All administrative steps should be carefully planned so that a blockage in one activity is identified and resolved with the lowest possible impact on the critical path. Time and resources are needed to streamline these processes but are not always available or considered a priority. Scientific advice obtained from stringent regulatory agencies about the minimum requirements for the study design, as well as necessary additional studies or pharmaceutical product development steps, will help to provide a clean dossier and facilitate the approval timeline. A simple but comprehensive dossier is always key to success.

The second element needed to rapidly complete a study that will give relevant results is a thorough epidemiological assessment before enrolling patients. During the development of fexinidazole, the scarcity of cases and steady reduction in numbers of cases made it necessary to locate the most endemic areas and upgrade existing health structures so that patients would be found and included in the trial. During the trials, slow recruitment required the opening of new trial sites to adhere to the planned timelines as closely as possible. This problem is specific to an increasingly rare disease such as HAT, but other treatments may also be affected if the preliminary assessment does not carefully consider the inclusion and exclusion criteria, as not every case of a disease will be eligible to participate in a clinical trial. Specific attention needs to be given to women of reproductive age and the feasibility of protecting them from unwanted pregnancies during the trial. ^18^

## CONCLUSION

It is not straightforward to generalize the experience of developing a new drug for HAT to other interventions, as many difficulties and risks will be specific, both epidemiologically and geographically. However, some elements can be generalized. Our experience has shown that it takes longer to gather funding, prepare proposals, and submit them to ethics and regulatory authorities than the actual execution of a development program. In addition, careful planning and engagement of key stakeholders from the start is critical. Anything that was not considered during the planning period will certainly delay your product development. Furthermore, unexpected challenges may delay even the best-laid plan, such as the disruption of introduction activities by the COVID-19 pandemic. Nonetheless, any effort to compress timelines is worthwhile as it will accelerate access to new or improved public health interventions to the populations that need them the most.
